# Editorial: Application of genomics in livestock populations under selection or conservation

**DOI:** 10.3389/fgene.2024.1363839

**Published:** 2024-01-24

**Authors:** Anupama Mukherjee, Zexi Cai, Sabyasachi Mukherjee

**Affiliations:** ^1^ ICAR-National Dairy Research Institute, Karnal, Haryana, India; ^2^ Center for Quantitative Genetics and Genomics, Aarhus University, Aarhus, Denmark

**Keywords:** livestock genomics, selection, conservation, WGS, GWAS, genotyping

Genomics is one of the newest branches of biology that has progressed tremendously during the last decades. Genomics deals with the molecular structures, functions, evolution, and mapping of the genomes of any species and has significantly generated new information that has improved our understanding of the complex biology and genetic mechanisms of animal production systems. The advancement of genomics is linked with a number of key developments, which include the rapid expansion of next-generation sequencing and chip-based genotyping assays. Large-scale genomics data are now utilized more and more due to the dwindling cost of such sequencing and genotyping techniques. Livestock breeding programs, including selection and conservation efforts, have attained huge success due to affordable genomic prediction, particularly in dairy cattle. It is expected that there will be further reduction in the cost of these high-throughput genomic data generation platforms and more development of precise estimation methodologies. Multi-disciplinary involvement is going to further benefit the genomics community with the advancement of robust and reliable tools in the field of bioinformatics and their use in livestock breeding.

Keeping these developments in the area of livestock genomics in mind, the present Research topic of the Frontiers in Genetics titled “Application of Genomics in Livestock Populations under Selection or Conservation” was aptly selected with several major themes that highlighted the usage of genomics for conservation, current methods of genomics, application of whole-genome- and genome-wide-based techniques, and use of different bioinformatics tools and pipelines for the processing of genomic data. The resulting efforts contributed to the publication of a total 19 research papers in the current volume, comprising major focal points in the area of genomics of livestock and other species with the concerns of the present day. However, the ocean of genomics is too vast, and even this wide-array of published articles could hardly justify an ounce of that vastness!

Nonetheless, genuine efforts were made to include articles in this volume on those central themes of genomics that comprise the major skills and techniques employed in various animal populations for selection and conservation issues. These include genome-wide association studies (GWAS), differential gene expression utilizing transcriptome data, and analysis of selection signatures through whole-genome sequencing and high-density genotyping datasets, which are utilized for discovering genes and genomic variants that control significant traits of importance in livestock species.

Terefe et al. in African cattle, Naji et al. in Austrian and Chinese cattle, Karam et al. in sobaity seabream, Heidaritabar et al. in crossbred and purebred pigs, Li et al. in yak populations, Chen et al. in buffaloes, and Liu et al. in Chinese Qaidam cattle utilized whole-genome-sequenced datasets for the analysis of variant calling, selection signatures, and genomic copy number variations to identify genes related to major economic traits of importance, including production, growth, immunity, and adaptability, in these livestock species. Terefe et al. provided possible examples of convergent selection between cattle and humans through the identification of unique selection signatures in African cattle living in the Ethiopian highlands.

Liu et al. generated skin transcriptome data from cashmere goats and identified key pathways and six hub genes (*PDGFRA, WNT5A, PPP2R1A, BMPR2, BMPR1A*, and *SMAD1*) that regulate cashmere development in these goats that are mediated by exogenous melatonin. This study is expected to provide a foundation for understanding the mechanism of melatonin-regulated cashmere growth. Liu et al. identified LTBP2 gene polymorphisms and their association with the thoracolumbar vertebrae number, body size, and carcass traits in Dezhou donkeys, which will be useful as a molecular marker to improve the production performance in this donkey population.

Genomic selection (GS) is another potential breeding tool that can reduce the generation interval, improve the accuracy of selection, and bring genetic improvement and, therefore, has been successfully employed in many farm animals for more than a decade now (Hayes et al., 2009; Gorjanc et al., 2015; de Koning, 2016; Meuwissen et al., 2016; Wiggans et al., 2017; Yang et al., 2020). Ning et al. studied various marker densities and designed several statistical models to increase the accuracy of genomic selection for wool traits in Angora rabbits. They are the first to estimate genomic heritability in Angora rabbits and showed that their work will be able to provide key strategies to optimize GS using 50k marker density in rabbits for early selection of various wool traits. Wiggans and Carrillo reviewed the progress of GS in the United States and found that the dairy genomic selection program has doubled the rate of annual genetic gain since 2010, with a rapid increase in the number of genotype evaluations for over 50 traits. The use of genomic information has enabled us to determine the value of animals at a much earlier age and has contributed to a dramatic increase in the rate of genetic improvement.

Culver and Labow (2002) mentioned that genomics is a multi-disciplinary field of biology that focuses on the structure, function, evolution, mapping, and editing of genomes. Unlike genetics, which refers to the study of individual genes having a role in inheritance and variation, genomics targets the combined characterization of all of an organism’s genes, their combined inheritance, and variation on the organism (WHO, 2020). Genomics also includes studies of various within-the-genome phenomena such as epistasis (the effect of one gene on another), pleiotropy (one gene affecting more than one trait), heterosis or hybrid vigor, and the different interplay of loci and alleles within the genome (Pevsner, 2009). Since the domain of genomics is quite broad, it is unlikely that the coverage of the present Research Topic will be able to encompass all. Nonetheless, the 19 articles did cover a great number of aspects of genomics and their application in various animal populations for selection and conservation. Given that these articles are interesting and timely, we agree that there is still scope for improvement in incorporating genomics Research Topic for the selection of complex traits, i.e., the selection for traits with low heritability and disease resistance in farm animals and other species. High-throughput phenotyping or phenomics and precision farming are such tools that are being applied along with genomics in most advanced countries for livestock breeding programs (Pedrosa et al., 2023). We sincerely hope that a future Research Topic on genomics may well embrace many other emerging areas within genomics, i.e., phenomics, epigenomics, and metagenomics, utilized not only for genetic improvement programs but also for the sustainability of livestock production systems.

## References

[B1] Culver K. W. Labow M. A. (2002). “Genomics,” in Genetics. Editor Robinson R. (New York, NY: Macmillan Science Library, Macmillan Reference USA).

[B2] de Koning D. J. (2016). Meuwissen et al. on Genomic Selection. Genetics 203 (1), 5–7. 10.1534/genetics.116.189795 27183561 PMC4858795

[B3] Gorjanc G. Cleveland M. A. Houston M. D. Hickey J. M. (2015). Potential of genotyping-by-sequencing for genomic selection in livestock populations. Genet. Sel. Evol. 47, 12. 10.1186/s12711-015-0102-z 25887531 PMC4344748

[B4] Hayes B. J. Bowman P. J. Chamberlain A. J. Goddard M. E. (2009). Invited review: genomic selection in dairy cattle: progress and challenges. J. Dairy Sci. 92 (2), 433–443. 10.3168/jds.2008-1646 19164653

[B5] Meuwissen T. H. Hayes B. Goddard T. (2016). Genomic selection: a paradigm shift in animal breeding. Anim. Front. 6, 6–14. 10.2527/af.2016-0002

[B6] Pedrosa V. B. Boerman J. P. Gloria L. S. Chen S. Y. Montes M. E. Doucette J. S. (2023). Genomic-based genetic parameters for milkability traits derived from automatic milking systems in North American Holstein cattle. J. Dairy Sci. 106, 2613–2629. 10.3168/jds.2022-22515 36797177

[B7] Pevsner J. (2009). Bioinformatics and functional genomics. 2nd Edn. Hoboken, NJ: Wiley-Blackwell.

[B8] WHO (2020). WHO definitions of genetics and genomics. World Health Organization, 20. Archived from the original on December.

[B9] Wiggans G. R. Cole J. B. Hubbard S. M. Sonstegard T. S. (2017). Genomic selection in dairy cattle: the USDA experience. Annu. Rev. Anim. Biosci. 5, 309–327. 10.1146/annurev-animal-021815-111422 27860491

[B10] Yang C. J. Sharma R. Gorjanc G. Hearne S. Powell W. Mackay I. (2020). Origin specific genomic selection: a simple process to optimize the favorable contribution of parents to progeny. G3 Genes| Genomes| Genet. 10 (7), 2445–2455. 10.1534/g3.120.401132 PMC7341124 32430306

